# Understanding Autoimmune Diabetes through the Prism of the Tri-Molecular Complex

**DOI:** 10.3389/fendo.2017.00351

**Published:** 2017-12-14

**Authors:** Matthew L. Bettini, Maria Bettini

**Affiliations:** ^1^Pediatric Diabetes and Endocrinology, Baylor College of Medicine, Texas Children’s Hospital, McNair Medical Institute, Houston, TX, United States

**Keywords:** T cell, autoimmunity, type 1 diabetes, human leukocyte antigen, regulatory T cell, thymic selection

## Abstract

The strongest susceptibility allele for Type 1 Diabetes (T1D) is human leukocyte antigen (HLA), which supports a central role for T cells as the drivers of autoimmunity. However, the precise mechanisms that allow thymic escape and peripheral activation of beta cell antigen-specific T cells are still largely unknown. Studies performed with the non-obese diabetic (NOD) mouse have challenged several immunological dogmas, and have made the NOD mouse a key experimental system to study the steps of immunodysregulation that lead to autoimmune diabetes. The structural similarities between the NOD I-A^g7^ and HLA-DQ8 have revealed the stability of the T cell receptor (TCR)/HLA/peptide tri-molecular complex as an important parameter in the development of autoimmune T cells, as well as afforded insights into the key antigens targeted in T1D. In this review, we will provide a summary of the current understanding with regard to autoimmune T cell development, the significance of the antigens targeted in T1D, and the relationship between TCR affinity and immune regulation.

## Introduction

**Figure 1:**
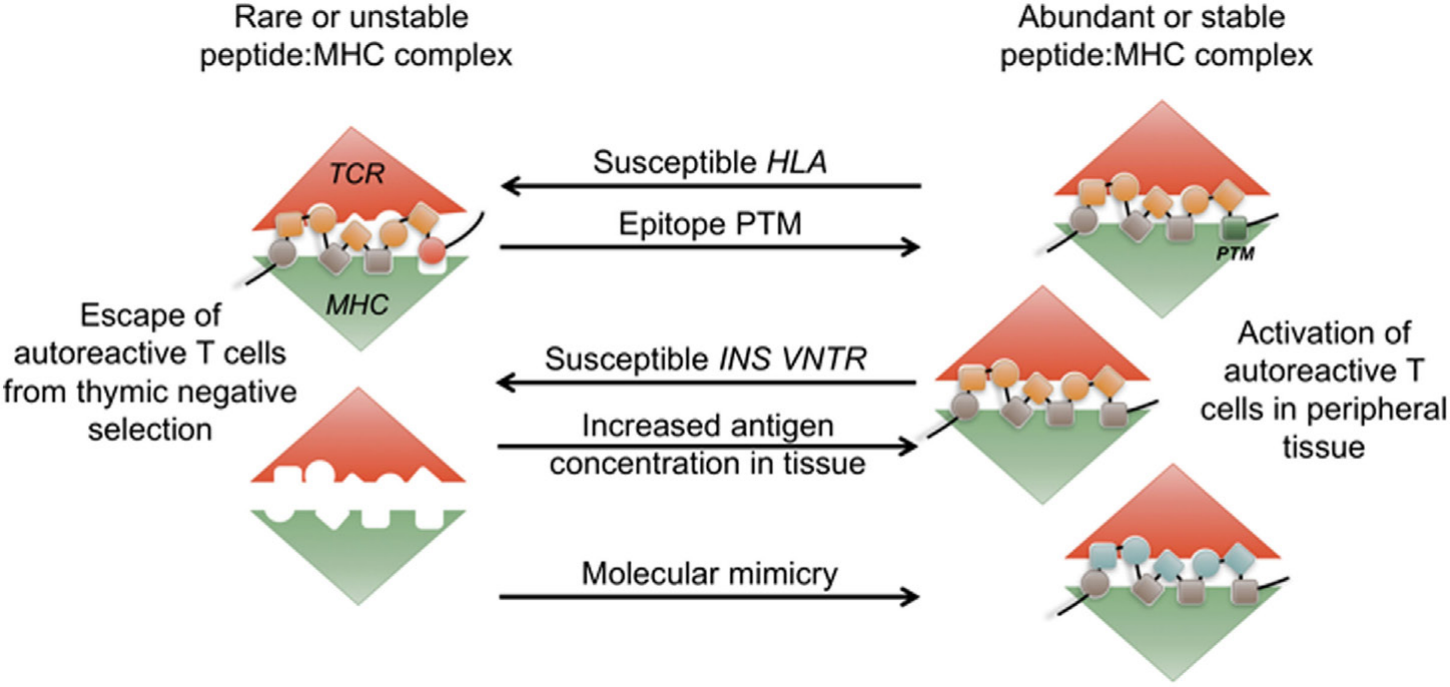
Abundance and stability of the tri-molecular complex at the interface of tolerance and autoimmunity. During thymic development, rare or unstable self-peptide: major histocompatibility (MHC) complexes can lead to escape of autoimmune T cells. Human leukocyte antigen (HLA)-DQ8 and H2-IA^g7^ susceptible alleles form unstable complexes with insulin epitope B:9-23. *INS-VNTR* susceptible allele results in lower level of insulin presentation in the thymus. Post-translational modifications (PTM) of self-epitopes can lead to more stable complexes in periphery. Increase in antigen availability in periphery or presence of structurally similar peptides in the context of infection (molecular mimicry) leads to priming of autoimmune T cells.

Autoimmunity is generally associated with polygenetic susceptibility, while the initial precipitating event is likely triggered by an environmental stressor (1–4). The major alleles associated with most autoimmune disorders are the human leukocyte antigen (HLA), and several alleles are shared among autoimmune conditions (5–8). This suggests that a common T cell-dependent mechanism is the underlying cause of tissue-specific autoimmunity irrespective of the organ or tissue being targeted. Although several hypotheses have been put forth to explain the HLA-mediated susceptibility, the exact mechanisms are still largely unknown. HLA structure selects for a particular peptide sequence motif and can affect the stability of the peptide:HLA complex (9). It is likely that autoimmune epitopes are not efficiently presented within the susceptible HLA molecules during thymic selection, or alternatively are presented with increased stability or at a higher concentration in the target tissue (10). Clearly, HLA allele structure is not the only parameter that might affect the stability of the tri-molecular complex [T cell receptor (TCR)/HLA/peptide], and not all individuals with T1D possess susceptible HLA alleles. Lower level of tissue antigen expression in the thymus, the relative abundance of self-antigen at the tissue site, an increase in immunogenicity of self-peptides either *via* post-translational modifications (PTMs) or molecular mimicry could all influence the stimulatory capacity of peptide:HLA complexes in periphery (Figure 1). How these changes in epitope immunogenicity could affect disease development will be discussed in this review.

The spontaneously diabetic non-obese diabetic (NOD) mouse model has been a useful system for identification of the key mechanisms important in the development of autoimmunity due to its significant similarity to human T1D (11, 12). Nearly 6 years after HLA was first associated with T1D in humans (13, 14), the spontaneously generated NOD diabetic strain was obtained by the Jackson Laboratory from CLEA Japan, where it quickly became an invaluable tool in the etiology of T1D (11, 15). The importance of the major histocompatibility (MHC) locus was originally traced by congenic approach, where MHC locus was introgressed onto the NOD background (16, 17). Further analysis of mice that received a non-NOD MHC class II transgene confirmed the important contribution of I-A^g7^ to diabetes susceptibility (18). Although MHC II confers most of the susceptibility, there are over 50 genetic loci that make up the NOD diabetic phenotype (19). The polygenetic susceptibility of the NOD mouse strain mirrors human disease, and further underlies the complexity of T1D (20). Importantly, the I-A^g7^ MHC II variant has structural similarities with human susceptible DQ8 (DQA1*0301/DQB1*0302) (9, 21, 22). Moreover, many of the antigens targeted in autoimmune diabetes are shared between the two species (19). The similarities of the shallow and positively charged peptide-binding groove characteristic of both human DQ8 and mouse I-A^g7^, and significant concordance in antigenic targets have made it possible to uncover sequence characteristics of autoimmune epitopes that are relevant to human disease (23, 24). Nevertheless, the precipitating events that lead to T cell priming and beta cell destruction remain unclear (4, 25). While the NOD mouse model has been a prolific tool for mechanistic insight into the many facets of T1D pathogenesis, recent expansion of HLA-humanized mouse models now allow direct interrogation of human autoimmune tri-molecular complex (TCR/HLA/peptide) and its role in loss of self-tolerance.

## Evidence for T Cell-Mediated T1D

A large body of evidence accumulated over several decades has implicated beta cell-specific immune response and, in particular, beta cell-specific T cells as the main drivers of autoimmune tissue damage and development of T1D (12, 26, 27). Progression to disease in humans is associated with islet antigen-specific antibody responses, and T cells specific to islet antigens are found at higher frequencies in T1D patients (28–31). Importantly, both CD4 and CD8 T cells were observed directly in the pancreatic lesions, and islet antigen-specific T cells have been cloned from pancreatic islets of T1D organ donors (32–38). HLA, being the major risk allele, implies that inherent structural differences in HLA and, consequently, TCRs selected on those HLA alleles lead to erroneous T cell reactivity to self (5, 39, 40). While class II HLA alleles confer the majority of the genetic susceptibility, certain class I alleles have been shown to impose a separate risk (41). Multiple antigens are targeted by both CD4 and CD8 T cells in T1D. Beta cell-specific antigens presented by Class II molecules include preproinsulin (PPI), insulinoma-associated antigen (I-A2), glutamic acid decarboxylase (GAD) 65, heat shock protein (HSP)-60, HSP-70, islet-specific glucose-6-phosphatase catalytic subunit-related protein (IGRP), and zinc transporter (ZnT8) (42–44). While MHC class I responses display similar wide range of antigenic targets, including PPI signal peptide, IA2, ZNT8, human islet amyloid polypeptide (IAPP), IGRP, and GAD65 (45). The progression to T1D in humans is associated with accumulation of islet antigen antibody reactivity to IAA, GAD65, IA-2, and ZnT8, which mirrors the intra- and inter-molecular “antigenic spread” of T cell responses (46, 47). Other non-HLA allelic risk variants are associated with pathways involved in T cell development, activation, and function, further highlighting the importance of T cells are the key drivers of autoimmunity (19).

## HLA Mechanisms of Autoimmunity

While the precise mechanisms that lead to loss of tolerance are multifaceted, HLA-DQ8 susceptibility implies that the stability of the tri-molecular complex is an important aspect that underlies autoimmune T cell responses (Figure 1). The inbred NOD mouse model that possesses a single susceptible MHC class II allele I-A^g7^ (I-A^d^α/I-A^g7^β) has played a vital role in uncovering the mechanisms involved in the development of T1D. The structural similarities characterized by the shallow peptide-binding groove and the positive charge in the p9 peptide-binding pocket present in both I-A^g7^ and human HLA-DQ8 point to a similar mechanism of autoimmune susceptibility (22). The potential mechanisms include altered thymic selection due to peptide:MHC instability, and/or preferential binding and presentation of beta cell neo-antigens formed *via* post-transnational modifications in the periphery (Figure 1). Biochemical analyses revealed a propensity for both DQ8 and I-A^g7^ to bind peptides with negatively charged C-terminus (48). In the case of celiac disease, which is also associated with DQ8 susceptibility, gluten peptides targeted in disease have a negative charge at the C-terminus, which results in their stable binding to DQ8 (49). Although this observation suggests that key epitopes targeted in T1D should similarly contain negatively charged residues at p9, most beta cell antigenic epitopes lack this trait. Moreover, the dominant insulin epitope B:9-23 has a positively charged arginine at the C-terminus. Nevertheless, in support of this hypothesis, a mutation of InsB_9–23_ at presumptive p9 to a negatively charged glutamic acid increased the immunogenicity of the epitope and augmented the activation of insulin-specific T cells (50). In addition, a recent study has identified IAPP and Chromogranin A (ChgA) epitopes in beta cells that have been modified by peptide fusion to acquire a negative charge at the C-terminus (35). The modified peptides were significantly more immunogenic compared to unmodified wild-type epitopes. This groundbreaking finding offered a potential explanation for lack of efficient thymic selection under conditions of unstable tri-molecular complex formation in the thymus, followed by priming and activation of autoreactive T cells in response to modified and stable peptide:MHC complexes in peripheral tissue.

## Thymic Development of Autoreactive T Cells—What is the Evidence for Altered Thymic Selection in Autoimmunity?

**Figure 2:**
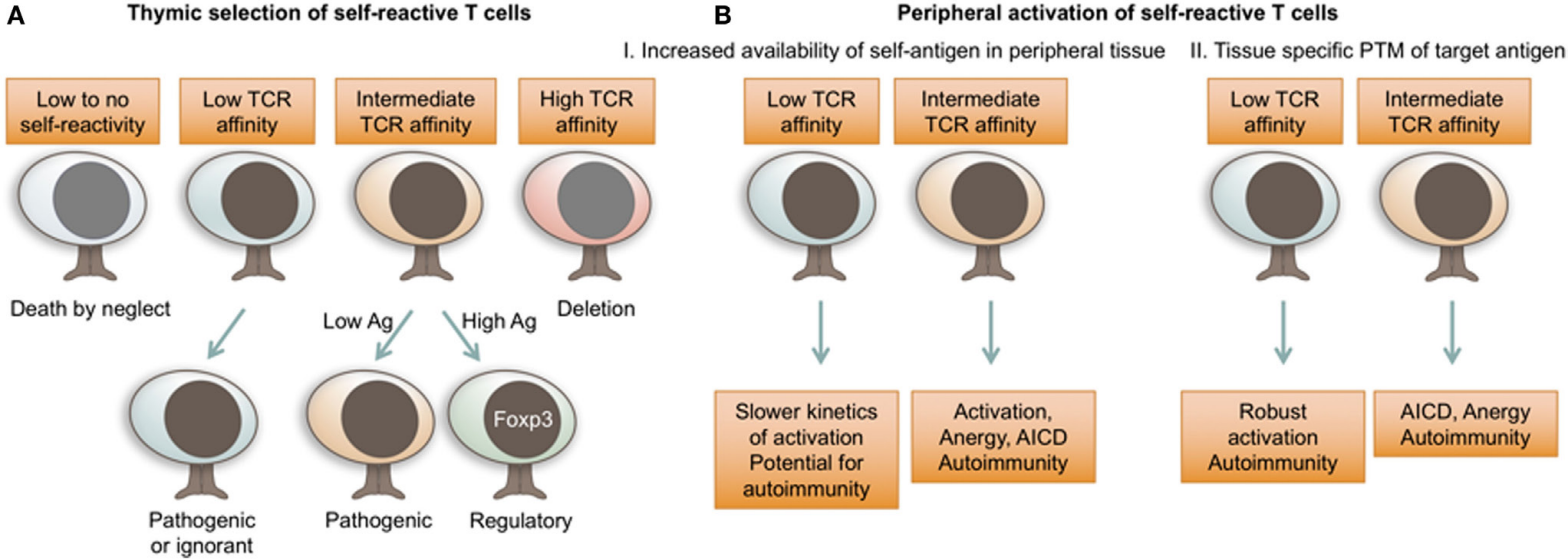
T cell receptor (TCR) affinity for self dictates autoimmune T cell fate decisions. **(A)** TCR affinity for self-ligands and antigen availability dictate thymocyte fate choices during thymic selection. While autoimmune T cells can be selected with a range of TCR affinities, increased antigen availability and relatively stronger self-reactivity will preferentially result in the development of regulatory Foxp3^+^ T cells. **(B)** In peripheral tissues, self-reactive T cells are activated in response to increased concentrations of tissue antigen or highly immunogenic PTM antigens. While autoimmune T cells can possess a range of TCR affinities for self-antigen, lower affinity TCRs are less susceptible to peripheral mechanisms of tolerance. Ag, antigen; AICD, activation induced cell death; PTM, post-translational modification.

A body of evidence suggests an important role for altered thymic selection in the development of autoimmunity. Negative selection of autoimmune lymphocytes depends on sufficient amount of self-antigen available for presentation in the thymus, which is regulated by intra-thymic and extra-thymic sources, genetic variation in tissue antigen promoters, and effective antigen presentation on certain HLA alleles (Figures 1 and 2A). Normally, tissue-specific antigens are presented by *Autoimmune regulator* (*Aire*) and *Fezf2* expressing thymic medullary epithelial cells (mTECs) to aide in the deletion of self-reactive thymocytes (51, 52). MTECs can also transfer antigens, including beta cell antigens, to thymic resident dendritic cells (DCs), which in turn delete self-reactive T cells (53). Both DCs and *Aire* expressing mTECs are also essential in generating thymically derived Foxp3^+^ regulatory T cells (Tregs), a critical population for the establishment and maintenance of self-tolerance (54, 55). Indeed, there appears to be a correlation between a reduction in DC numbers and residual β cell function in T1D subjects (56), while the NOD mouse exhibits an overall reduction in DCs (57, 58). These observations suggest a relationship between self-tolerance and the absolute number of DCs present in the thymus and periphery. However, not all peripheral antigens are expressed by mTECs and, therefore, negative selection must also rely on peripheral antigen retrieval and delivery to the thymus by DCs. Importantly, studies have shown that the generation of thymic regulatory T cells by antigen-presenting mTECs and DCs early in life (neonatal) is critical in maintaining tolerance to self (55, 59). The idea of peripheral antigen exposure generating tissue-specific Tregs was elegantly demonstrated by Scharschmidt et. al., where skin colonization of *S. epidermidis* allowed for the development and trafficking of microflora-specific Tregs to the skin. Using sphingosine-1-phosphate receptor antagonist, FTY720, the authors blocked Treg egress and pinpointed the thymus as the main source for Treg development (59).

There is no direct evidence for thymic selection deficiencies in individuals with T1D; however, several key observations suggest that there is a role for altered selection in the development of autoimmune responses to insulin. The level of thymic insulin expression in humans is controlled in part by the polymorphic variable number of tandem nucleotide repeats found in the region proximal to the promoter region of the insulin gene (*INS*–VNTR) (60). It has been shown that VNTR I alleles express 26–63 tandem repeats while the VNTR III carries 141–209 repeats. This difference translates into higher thymic transcript levels for the VNTR III individuals and a threefold to fourfold relative protection from T1D (61, 62). It appears that the number of repeats affects AIRE binding to the insulin promoter region, thus controlling transcriptional regulation of insulin in the thymus (51, 62, 63). In support of alterations in thymic selection, analysis of human peripheral blood from T1D patients and healthy controls revealed that subjects expressing the *INS*-VNTR I (T1D-predisposing) allele displayed elevated frequencies of high affinity proinsulin-specific T cells compared to *INS*–VNTR I HLA–DR4 subjects (64). *INS*-VNTR allelic expression appears to determine insulin reactivity rather than the total number of insulin-reactive T cells, as both VNTR I and VNTR III groups displayed similar total number of insulin-reactive T cells in peripheral blood (64). However, it has only been hypothesized that the differences in thymic insulin expression between VNTR I and VNTR III subjects influence positive and negative selections of insulin-reactive T cells, but this has never been formally demonstrated *in vivo* due to a lack of VNTR mouse models.

The role of thymic insulin expression in the establishment of central tolerance has been addressed in the NOD mouse model by both deletion and overexpression of insulin in the thymus. Deletion of insulin specifically in thymic *Aire* expressing mTECs enhanced diabetes development in both male and female mice (65). In addition, transgenic overexpression of proinsulin, but not GAD65 (66) or IGRP (67), significantly delayed (68) or prevented (69) diabetes progression in NOD mice. However, in these studies overexpression of insulin was targeted to all MHC class II expressing APCs and, therefore, the relative role of central compared to peripheral tolerance was not determined (68, 69). A more recent set of experiments determined that a narrow window of ectopic proinsulin expression in APCs (from birth until weaning) could prevent the development of diabetes in NOD mice (70). This timeframe fits with a previous study that showed organ specific autoreactive T cell escape from the thymus is greatest during the first 10 days of life in NOD mice (71). In the former study by Jhala et. al., protection was due in part to the deletion of insulin-specific T cells, but also the inability of the remaining insulin-specific T cells to respond to cognate antigen in periphery (70). In our recent study, we tested two TCRs (4-8 and 12-4.1, Table 1) with defined affinities for InsB_9–23_ for their ability to escape negative selection in the presence of ectopic overexpression of insulin. Surprisingly, we did not observe any increase in thymic deletion of the relatively high (4-8) or low (12-4.1) TCRs, although the increase in insulin expression did protect mice from developing autoimmune diabetes. Protection from disease appeared to be due to an increase in Treg development with a significant increase in thymic, splenic, and pancreas-residing insulin-specific Tregs (72). These findings pose an intriguing possibility that the amount or stability of self-peptide:MHC complexes during thymic selection is more important for Treg development rather than deletion of self-reactive T cells (Figure 2A).

**Table 1:** Pathogenicity of beta antigen-reactive T cells.

T cell receptor	Restriction	Epitope	Model	Infiltration	% Diabetes	Reference
**Mouse**						

**Chromogranin A (ChgA)**
BDC2.5	IAg7	ChgA 359–372	Tg/Rg	Insulitis	75/100	(83, 96)
BDC10.1	IAg7	ChgA 359–372	Rg	Insulitis	100	(83)
**Insulin**						
12.4-1	IAg7	InsB 9–23	Tg/Rg	Insulitis	5/50/72	(82, 83, 103, 104)
12.4-4	IAg7	InsB 9–23	Rg	Insulitis	51	(82)
12.4-4m1	IAg7	InsB 9–23	Rg	Peri-insulitis	–	(82)
8-1.1	IAg7	InsB 9–23	Rg	Insulitis	27	(82)
P2	IAg7	InsB 9–23	Rg	No	–	(82)
1-10	IAg7	InsB 9–23	Rg	Peri-insulitis	48	(82)
4-8	IAg7	InsB 9–23	Rg	Insulitis	59	(82)
3-4	IAg7	InsB 9–23	Rg	Insulitis	21	(82)
G9C8	Kd/Db	InsB 15–23	Tg	Mild insulitis	–	(105)
2H6	IAg7	InsB 9–23	Tg	Prevents diabetes	–	(97)
8F10	IAg7	InsB 9–23	Tg	Insulitis	100	(100)
**Glutamic acid decarboxylase (GAD)**
PA17.9G7	IAg7	GAD65 284–300	Rg	no	–	(83)
PA15.14B12	IAg7	GAD65 206–220	Rg	no	–	(83)
PA19.5E11	IAg7	GAD65 206–220	Rg	Peri-insulitis	–	(83)
PA18.10E1	IAg7	GAD65 524–538	Rg	n/d	–	(96)
PA18.9H7	IAg7	GAD65 524–538	Rg	Peri-insulitis	–	(83)
IA4	IAg7	GAD65 217–236	Rg	Peri-insulitis	–	(83)
**Protein tyrosine phosphatase-like (IA2)**
Phogrin 13	IAg7	IA2 640–659	Rg	Peri-insulitis	–	(83)
Phogrin 18	IAg7	IA2 755–777	Rg	Mild insulitis	–	(83)
10.23	IAg7	IA2 676–688	Rg	Peri-insulitis	–	(83)
**Iselt-specific glucose-6-phosphatase (IGRP)**
8.3	Kd	IGRP 206–214	Tg	Insulitis	33	(95)
**Islet amyloid polypeptide (IAPP)**
BDC6.9	IAg7	DLQTLAL-NAAR (Ins-IAPP fusion)	Tg/Rg	Insulitis	56	(35, 83)
**Unknown islet antigen**
NY4.1	IAg7		Tg/Rg	Insulitis	72/60/71	(83, 95, 96)
AI4	Db		Tg	Insulitis	100	(98, 102)

**Human**						

**Glutamic acid decarboxylase (GAD)**
164	DR4	GAD65/67 555–567	Tg	Insulitis	–	(101)
T1D4	DR4	GAD65 115–127	Rg	Mild to no insulitis	–	(99)

Chromogranin A is the only other currently known beta cell antigen necessary for the initiation of autoimmune diabetes in NOD mice; however, expression of ChgA in the thymus has not yet been detected (73). Therefore, tolerance to ChgA may rely in part on transport of antigen by peripheral DCs to the thymus. Whether islet-derived antigens are carried to the thymus to promote islet-specific Treg development has not been explored; nevertheless, the divergent TCR repertoire between islet-infiltrating effector and regulatory T cells suggests a lack of local Treg conversion in favor of thymic lineage being the predominant Treg population in the pancreas (74). The thymic Treg niche was thought to be highly specialized and restricted (75); however, a recent study has demonstrated that the manipulation of either the number of antigen-presenting cells or an increase in antigen exposure within the thymus can expand the Treg niche (54).

While highly self-reactive T cells are removed from the T cell repertoire by negative selection, the quality and the quantity of self-reactive Tregs that develop from the moderately self-reactive thymocyte pool is a critical component of peripheral self-tolerance (Figure 2A). This idea is consistent with the observation that healthy individuals possess significant frequencies of self-reactive T cells, but are free from autoimmunity (76, 77). The escape of self-reactive T cells in itself is not just a byproduct of Treg development, but seems to serve an important immunological purpose, since some level of self-reactivity is associated with enhanced responsiveness to foreign pathogens (78–80). It is likely that the fine balance between beneficial self-reactivity and self-tolerance is uniquely perturbed in individuals with a susceptible genetic background. A slight change in thymic antigen expression or the overall stability of the tri-molecular complex could shift the T cell development spectrum toward Treg insufficiency, rather than escape of higher affinity cells. Therefore, the ratio of beta cell antigen-reactive Tregs vs. effector or memory T cells might be a better predictive biomarker of autoimmunity than the overall frequencies of self-reactive cells.

## TCR Parameters of T Cell Pathogenicity

Autoimmune T cell responses in general, as well as, the T cell population that infiltrates the NOD pancreatic islets, are composed of cells with different T cell lineages, diabetogenic or regulatory capabilities, antigenic specificities, and TCR affinities (10, 81–83). All of these parameters are directly influenced by the TCR (84). Therefore, TCR sequence, specificity, and affinity hold the key to understanding the dynamics of diabetogenic T cell responses during chronic progressive autoimmune disorders, such as T1D. The antigenic specificity of each TCR is dictated by the highly variable CDR3 region found within the α and β chains of the TCR heterodimer. The variability is the result of random genetic recombination events that bring together one of many variable (V) genetic segments with a joining (J) region. The large number of TCR sequences infiltrating an organ, their variability among individuals, and the heterodimeric structure of the TCR has been a significant roadblock in a comprehensive functional analysis of TCRs. In this section, we will summarize the studies that have investigated beta cell-specific TCR parameters for their ability to predict T cell pathogenic potential.

### TCR Sequence As a Biomarker of Pathogenicity

One of the main hurdles in the identification and functional analysis of beta cell-reactive T cells in humans with T1D is the breadth of antigens and epitopes that are targeted among affected individuals. Peptide/MHC tetrameric reagents have been the most effective approach to identify T cells with autoimmune potential; however, beta cell-reactive cells comprise a small population of peripheral blood, which makes such approaches technically challenging. Moreover, tetramers detect only the highest affinity subpopulation of T cells specific for a particular epitope, while the majority of autoimmune responder T cells are often overlooked, as was effectively demonstrated in the mouse model of multiple sclerosis (85). As such, the field is currently lacking sufficient approaches to perform in depth tracking of antigen-specific T cells over time. Recent technological advances in high-throughput sequencing have opened new avenues for tracking self-reactive T cells and could be easily applicable to studies of human tissue-infiltrating T cells (86). A promising biomarker approach could be based on high-throughput TCR sequencing with focus on TCR motifs known to be associated with a specific target epitope. While human CD4 responses have proven to be highly diverse (87, 88), CD8 T cells are generally more clonotypic (86). A recent study was able to identify a public CDR3 motif associated with IGRP_265–273_ specific memory T cells in antibody-positive subjects and individuals diagnosed with T1D (89). Their findings suggest that dominant clonotypes persist in the same individual over time, and some TCR sequences could be shared among individuals. Interestingly, the public TCR motif was also identified in healthy controls, although it was restricted to the naïve T cell compartment. While promising as a potential biomarker, such deep sequencing approaches necessitate knowledge of multiple TCR sequences associated with reactivity to several beta cell antigens.

### Antigen Specificity of Pathogenic TCRs

Although, T cells of multiple antigenic reactivities have been isolated from pancreatic islets of T1D donors (35–38), it does not necessitate that these cells are equally pathogenic or are actively involved in beta cell destruction. In order to identify potentially important initiating antigens in T1D, multiple beta cell proteins have been mutated on the NOD background, including IAPP, GAD65, insulin, IGRP, and islet Ag-2 (90–94). Interestingly, only the mutation of insulin and chromogranin resulted in protection against diabetes (73, 94). This suggests that insulin and chromogranin-reactive T cells are either critical for the initiation of autoimmunity, or are necessary for further propagation of the disease and the ultimate destruction of beta cells.

Over the years, pathogenic potential of T cells reactive to various islet antigens was directly assessed in single TCR systems. Multiple mouse and a few human TCRs reactive against various beta cell proteins have been expressed in mice utilizing both transgenic and retrogenic approaches (82, 83, 95–105) (Table 1). Importantly, the observed tissue infiltration and spontaneous disease development were highly variable among the antigenic specificities (Table 1). Single TCR mice expressing either insulin, chromogranin, or IGRP reactive mouse TCRs developed spontaneous diabetes, supporting the important pathogenic role for these reactivities in autoimmune diabetes. The majority of phogrin (IA2b) and I-A2 reactive mouse TCRs can induce islet infiltration, albeit without overt diabetes. Reactivity to multiple GAD epitopes, however, results in no disease and very limited infiltration for both human and mouse TCRs (Table 1). Based on these observations, it is likely that TCRs with select beta cell antigenic specificities are central to disease pathogenesis. Moreover, certain specificities might be important at different stages of disease, while others might not have a pathogenic but rather a regulatory effect, as was observed for GAD-reactive mouse T cells (106–109). Nevertheless, our ability to effectively extrapolate contribution of T cell specificities to disease in a polyclonal multi-antigen specific environment by analyzing their behavior in single TCR systems is limited. NOD mouse models exhibit a single MHC II restriction; therefore, pathogenic responses to antigens presented in alternative susceptible HLA class II or class I alleles might be overlooked. Alternatively, it is possible that inflammation induced by T cells specific for the initiating antigen results in exposure or modification of secondary antigens, leading to pathogenic activation of a distinct repertoire of T cells specific to the newly displayed epitopes.

The molecular determinants of pathogenic TCRs in autoimmunity are still largely unknown. Antigen availability, immunogenic modification of T cell epitopes, and TCR avidity could all shape the responses of beta cell-specific T cells (Figure 2). While it is still unclear whether antigen reactivity is an absolute pre-requisite for tissue entry, several experimental approaches have shown that T cell accumulation in NOD pancreatic islets is driven by antigen recognition (110–112). The difference in antigen availability could explain relative importance of T cell specificities in the development and progression of autoimmunity. For example, reduced pathogenicity of GAD65-reactive T cells in NOD mouse model might be due to insufficient antigen availability in the pancreas. T cell reactivity to GAD65 and GAD67 can be observed early in NOD mice (113, 114), and antibodies specific for GAD are associated with progression to T1D in humans (115), which suggests a role for GAD reactivity in T1D. However, relative to other beta cell antigens, GAD T cells exhibit reduced pathogenicity in mouse models compared to other antigens (Table 1), with only one study showing diabetogenic activity of GAD65-reactive T cells (116). The rather mild pathogenic potential of GAD-reactive T cells in NOD model could be attributed to relatively low levels of both GAD65 and GAD67 expressed in the mouse islets compared to rat or human pancreas (117). In support of this, overexpression of GAD65 under the rat insulin promoter enhanced pancreatic infiltration of GAD-reactive T cells (110). Although, this observation serves as a proof of principle for the importance of antigen availability for islet infiltration, overexpression of GAD65 in polyclonal NOD mice does not result in enhanced insulitis or diabetes (118). Therefore, other parameters in addition to islet antigen availability must regulate T cell pathogenic potential.

### TCR Affinity of Pathogenic T Cells

It is logical to assume that TCR affinity for antigen is associated with increased pathogenicity; however, that is not always the case, as we have shown for insulin-reactive TCRs. When eight NOD CD4 T cell-derived TCRs with variable affinity for insulin InsB_9–23_ epitope were compared for their ability to drive spontaneous diabetes, high- and low-affinity T cells were similarly pathogenic (82) (Table 1). This is consistent with observations that a polyclonal autoimmune T cell response can encompass a wide range of TCR affinities, and low-affinity T cells are important contributors to the immune response (85, 119). However, it appears that there are certain functional distinctions between high- and low-affinity insulin-reactive T cells. Compared to high-affinity TCRs, low-affinity TCRs were less sensitive to thymic negative selection pressures, exhibited lower frequencies of Foxp3^+^ T cell development, and had a reduction in negative regulators of T cell activation (82). Their inability to reach the threshold for engagement of regulatory elements could allow the low-affinity cells to exert effector functions and induce beta cell damage even under relatively low level of TCR stimulation (Figure 2B).

## Peripheral Priming of Autoimmune T Cells

### Molecular Mimicry

The mechanisms behind self-reactive T cell priming and ensuing loss of self-tolerance are complex and poorly understood. Autoimmune T cells exhibit a level of reactivity for self-antigens, but are somehow able to escape negative selection in the thymus. In the periphery, these cells encounter cognate self-antigen with enough affinity and in the right context to become activated and cause tissue damage. In the case of T1D, studies have implicated molecular mimicry as a potential trigger, where beta cell-reactive T cells could undergo initial priming and activation in response to structurally similar microbial epitopes (120) (Figure 1). While the direct evidence for molecular mimicry as a cause for autoimmunity is lacking, recent work exposing the previously unrecognized propensity of T cells for cross-reactivity reinforces molecular mimicry as a valid hypothesis (87, 121, 122). Islet-specific glucose-6-phosphatase catalytic subunit-related protein (IGRP)-reactive CD8 T cells were shown to recognize a transporter protein peptide of *Fusobacteria*. Importantly, activation of IGRP-specific NY8.3 T cells by *Fusobacteria* contributed to enhanced diabetes development (123). Microflora composition in general has been implicated in both human and mouse T1D. In mice, gender hormones influence microbiota and subsequent T1D development (124, 125), while autoantibody-positive children have distinct microbiota signatures (126). It has yet to be seen whether specific microbiota species drive activation of islet-reactive T cells leading to beta cell destruction.

### Unusual Orientation of the Tri-Molecular Complex

It is hard to reconcile exceedingly lower reactivity of autoimmune T cells to their cognate antigen, compared to non-self-reactive TCRs, with their capacity to exert significant tissue damage. For example, insulin-reactive TCR 12-4.1 isolated from pancreatic islets of NOD mice exhibits barely detectable reactivity to insulin *in vitro* (82), but causes spontaneous diabetes in 50–80% of mice (82, 103) (Table 1). As we alluded to earlier, lower affinity self-reactive TCRs are to some degree resistant to central and peripheral tolerance mechanisms, which might explain their ability to persist in an activated state (82). However, it is still unclear how self-reactive T cells with very low affinity for antigen are capable of causing beta cell destruction and highly penetrant diabetes. It is possible that the inherent unusual TCR structural and signaling characteristics are potential contributing factors that lead to unique responsiveness of autoimmune T cells. Crystal structures of autoimmune TCR:pMHC complexes have uncovered an unconventional docking of self-reactive TCRs on pMHC (127–129). Moreover, self-reactive human and mouse TCRs form unusual disorganized T cell synapses, exhibit slower kinetics of TCR signaling pathways, and yet they are still able to undergo activation and exert effector functions (130, 131). Conceivably, these characteristics allow autoimmune T cell escape from thymic selection, while in the target tissue high level of antigen is sufficient to elicit effector response.

### Tissue-Specific PTM of Target Epitopes

In the case of autoimmune T1D, beta cell fragility characterized by increased susceptibility to oxidative and ER stress may be a critical factor in loss of self-tolerance. A consequence of the cellular stress is the altered processing and changes in PTM of proteins. The changes in beta cell epitopes can lead to the generation of tissue-specific neo-antigens that are not expressed in the thymus. T cells specific for neo-antigens can evade mechanisms of central tolerance and initiate an autoimmune response once exposed to PTM antigens in periphery (Figure 1). Interestingly, insulin containing granules are highly immunogenic compared to artificially synthesized protein, which suggests some manner of PTM takes place within the NOD beta cell granules (10). In the case of the dominant insulin epitope targeted in the NOD mice (InsB_9–23_), the modification likely affects the MHC-binding residue of the peptide, resulting in stable binding of peptide in a register that is normally unstable and very likely presented at low levels in the thymus (10, 50, 132). In support of this idea, studies have shown that a mimotope of the InsB_9–23_ insulin peptide with a change in the MHC anchor residue (R22E) was highly stimulatory for insulin-reactive T cells, and R22E peptide:MHC tetramers identified insulin-reactive cells within the islet-infiltrating T cell population (50, 133). Just in the last few years, it has been demonstrated that neo-antigenic PTM epitopes can form by fusion of either ChgA or IAPP peptide with a pro-insulin peptide (35). These fusion peptides were highly stimulatory to IAPP- and ChgA-reactive diabetogenic NOD T cell clones, as well as CD4 T cells isolated from the islets of T1D donors (35, 37). While the fusion peptides were identified in beta cells, it is unknown whether their formation is increased during inflammation or ER stress. More recent work has identified immunogenic peptides generated from an alternate insulin reading frame, the translation of which was further increased under ER stress (134). CD8 T cell clones isolated from peripheral blood of T1D subjects and specific for these defective ribosomal products (DRiPs) were able to cause direct beta cell damage *in vitro*, supporting a potentially critical role for DRiPs in T1D. This is yet another PTM mechanism within a mounting evidence for connection between beta cell ER stress and generation of immunogenic PTMs. Nevertheless, it is still unknown exactly to what extent PMT antigen-specific T cells contribute to T1D.

At the moment, we have very little insight into the functional concentration of PTM antigens vs. wild type epitopes presented in the inflamed tissue, the relative frequency of PTM-reactive T cells vs. T cells that recognize the wild-type epitopes, or how these parameters change over the course of chronic autoimmune tissue damage. It is likely that some T cells have a restricted specificity to either PTM or wild-type antigens, while others respond to both with different levels of activation. Addressing these questions will lead to our better understanding of the triggers that induce autoimmune response, as well as identification of the initiating antigens and the key pathogenic T cell populations. It is currently unknown whether tissue-specific PTM antigens are transported and expressed in the thymus. In order to model how the presence of post-translationally modified peptides in the thymus could alter the selection of insulin-reactive TCRs (4-8 and 12-4.1) that normally escape negative selection, we ectopically expressed the R22E insulin mimetope in bone marrow-derived APCs (72). In the presence of R22E, the high-affinity 4-8 TCR bearing thymocytes were efficiently deleted, while the low affinity 12-4.1 population was affected to a lesser degree, albeit still showing an increase in negative selection based on Annexin V staining. Nevertheless, the ectopic expression of R22E significantly reduced peripheral T cells and halted any islet infiltration in both the 4-8 and 12-4.1 retrogenic mice. These results suggest that unlike expression of wild-type antigen, expression of PTM epitopes in the thymus results in efficient deletion of autoimmune T cells.

Accumulating evidence indicates PTMs as the key to our understanding of autoimmune disease development (35, 135–140). Importantly, T cells specific for PTM GAD65 and ChgA epitopes have been identified in individuals diagnosed with T1D (35, 139). Although the evidence so far is limited, PTM epitope expression is likely restricted to peripheral tissue and is absent from the thymus. While wild-type self-proteins presented in the thymus successfully limit development of high-affinity self-reactive T cells, lower affinity T cells evade central tolerance to be able to respond to PTM antigens in periphery (Figures 1 and 2B). Moreover, it is conceivable that the lack of PTM antigen expression in the thymus could lead to holes in the Treg repertoire. While multiple studies have shown that modification of beta cell epitopes increases their immunogenicity, it is unclear what proportion of antigens in the pancreas has been modified. Presumably, relatively low concentrations of immunogenic PTM epitopes are sufficient to prime autoimmune T cells, while presence of wild-type epitope is adequate for propagation of chronic autoimmune response. Further biochemical analyses of the pancreatic beta cells are necessary to identify the predominant PTM epitopes and the stress conditions that lead to their development.

## HLA-Humanized Mice to Model T1D Antigen Responses

While we have learned a great deal from the NOD mouse, there are certain limitations to the conclusions and parallels we can draw to human T1D. In order to improve the model, several HLA transgenic mouse strains expressing susceptible or protective alleles have been generated, some of these on the NOD background. Surprisingly, NOD mice expressing susceptible DQ8 or DR4 alleles do not develop spontaneous diabetes (141–143). However, HLA-DQ8 humanized mice do develop spontaneous autoimmune cardiomyopathy (144). Still, both DR4 and DQ8 alleles support the development of beta cell-reactive autoimmune T cells but require an additional trigger to initiate beta cell targeted autoimmunity. When DR4 and DQ8 mice were crossed with transgenic mice expressing B7.1 co-stimulatory molecule on beta cells, both HLA-humanized strains developed spontaneous diabetes (141). The main utility for HLA-humanized mice has been realized by performing systematic identification of the key antigenic epitopes presented on human HLAs (24). Future studies should be extended to assess the *in vivo* functional potential of human autoimmune TCRs specific for key immunogenic epitopes. To date only one beta cell antigen-reactive human TCR transgenic mouse with specificity for GAD65 has been described (101). *In vivo* functional analysis of TCRs, and human TCRs in particular, has been hindered due to limited access to patient samples, labor, and time involved in generating TCR transgenic mice. We have overcome the limitation of TCR transgenic system by utilizing a TCR retrogenic approach that allows rapid functional analysis of multiple TCRs through retroviral gene delivery (110, 145, 146). Using this approach, we have expressed a GAD65_115–127_ reactive TCR isolated from peripheral blood of an individual diagnosed with T1D (99). Although we observed robust development of GAD-reactive T cells in this system, similar to the transgenic expression, we detected a low level of islet infiltration. Future analyses should be expanded to other beta cell protein epitopes targeted in human T1D, including PTM epitopes. The humanized TCR retrogenic approach will allow efficient and relatively high-throughput analysis of autoimmune antigens important in human disease, and can be utilized as a platform for development of antigen-specific immunotherapies. It is likely that many questions pertinent to our understanding of autoimmune T cell development and pathogenicity will be eventually addressed in the context of human susceptible HLA alleles and human TCRs.

## Conclusion

The biology of low-affinity autoimmune T cells has been perplexing due to the seeming contradiction between suboptimal *in vitro* responses and robust *in vivo* pathogenicity. In many cases, self-reactive autoimmune T cells do not follow the dogma prescribed by studies performed with T cells specific for infectious or model antigens. In addition to unusual TCR:pMHC interactions and downstream signaling, autoimmune antigens themselves can have atypical characteristics. Over the years, it has become clear that antigens targeted in autoimmunity, and particularly in T1D, are often modified versions of self-peptides that are presented during thymic selection. These exceptions to the rule characteristic of autoimmune T cell responses are often centered on the stability of the tri-molecular complex as a master switch from tolerance to autoimmunity.

## Author Contributions

MB and MLB developed the concept, prepared the figures, and wrote the manuscript.

## Conflict of Interest Statement

The authors declare that the research was conducted in the absence of any commercial or financial relationships that could be construed as a potential conflict of interest.
